# Case Report: Cytokine storm syndrome causing retinal inflammatory factor storm

**DOI:** 10.3389/fimmu.2026.1835853

**Published:** 2026-05-12

**Authors:** Na Liu, Jingyi Bai, Gaixia Zhai, Shaopeng Wang

**Affiliations:** Department of Ophthalmology, Zibo Central Hospital, Shandong, China

**Keywords:** cytokine storm syndrome, interleukin-6, macrophage-like cell, retina, systemic inflammatory response syndrome

## Abstract

Cytokine storm syndrome (CSS) represents a severe systemic inflammatory condition precipitated by the hyperactivation of immune cells and the consequent excessive release of cytokines, triggered by various factors. This report details the case of a 23-year-old woman admitted with acute high fever and subsequently referred to the ophthalmology department due to blurred vision. Multimodal imaging documented the progression of inflammatory cytokine storm in her fundus, and intraocular fluid analysis revealed abnormal cytokine levels. Following the resolution of inflammation, ocular symptoms and signs showed marked improvement. This report describes ocular manifestations of cytokine storm syndrome, underscoring the susceptibility of the fundus to systemic immune dysregulation despite the presence of the blood-retinal barrier.

## Introduction

A myriad of pathogens, autoimmune disorders, malignant conditions, genetic abnormalities, and specific therapeutic interventions can induce life-threatening systemic inflammatory syndromes characterized by a massive cytokine release due to immune cell hyperactivation, a condition universally recognized as cytokine storm syndrome (CSS). CSS frequently manifests in the context of allergic reactions, graft-versus-host disease, acute respiratory distress syndrome (ARDS), and systemic inflammatory response syndrome (SIRS) (1, 2). Here, we present a case of a specific retinal manifestation caused by infection-associated cytokine storm syndrome, which is primarily characterized by retinal vasculitis involving retinal inflammatory response and an excessive immune response, with significantly elevated levels of pro-inflammatory cytokines.

## Case report

A 23-year-old female presented to the infectious diseases department with a week-long history of fever, peaking at 40.4°C, accompanied by diffuse painful maculopapular eruptions on both lower limbs, dry throat, fatigue, poor appetite, and dysuria. She had undergone laryngoscopy at a local hospital, revealing acute laryngitis and an upper respiratory tract fungal infection. Blood tests indicated leukopenia (WBC 2.3*10^9/L) and thrombocytopenia (PLT 98*10^9/L). The patient has a healthy past and denies a history of similar episodes. Upon admission, she exhibited a temperature of 39.3°C, poor mental state, generalized weakness, acute appearance, cervical lymphadenopathy, and pharyngeal redness and swelling. Comprehensive physical examination and CT scans of the head, chest, and abdomen revealed no significant abnormalities. Laboratory results showed that alanine aminotransferase (ALT), aspartate aminotransferase (AST), lactate dehydrogenase (LDH), C-reactive protein (CRP), homocysteine, and D-dimer were significantly elevated, accompanied by elevated levels of interleukin-6 (IL-6) and procalcitonin (PCT) and a decrease in absolute value of Natural killer (NK) cells in the blood, and serum electrolytes were abnormal (Table 1). Additional tests, including anti-O quantitative, rheumatoid quantitative, TB infected T cell spot, COVID-19 nucleic acid, tumor marker, EB virus, human immunodeficiency virus antigens and antibodies (HIV Ag/Ab), treponema pallidum specific antibody(syphilis), hepatitis B virus surface antigen (HBsAg), hepatitis B virus e antigen (HBeAg), CMV-DNA and TORCH tests, yielded normal results. Based on her medical history, clinical presentation, and laboratory findings, a preliminary diagnosis of fever of unknown origin, acute pharyngitis, and upper respiratory tract fungal infection was made.

**Figure 1:**
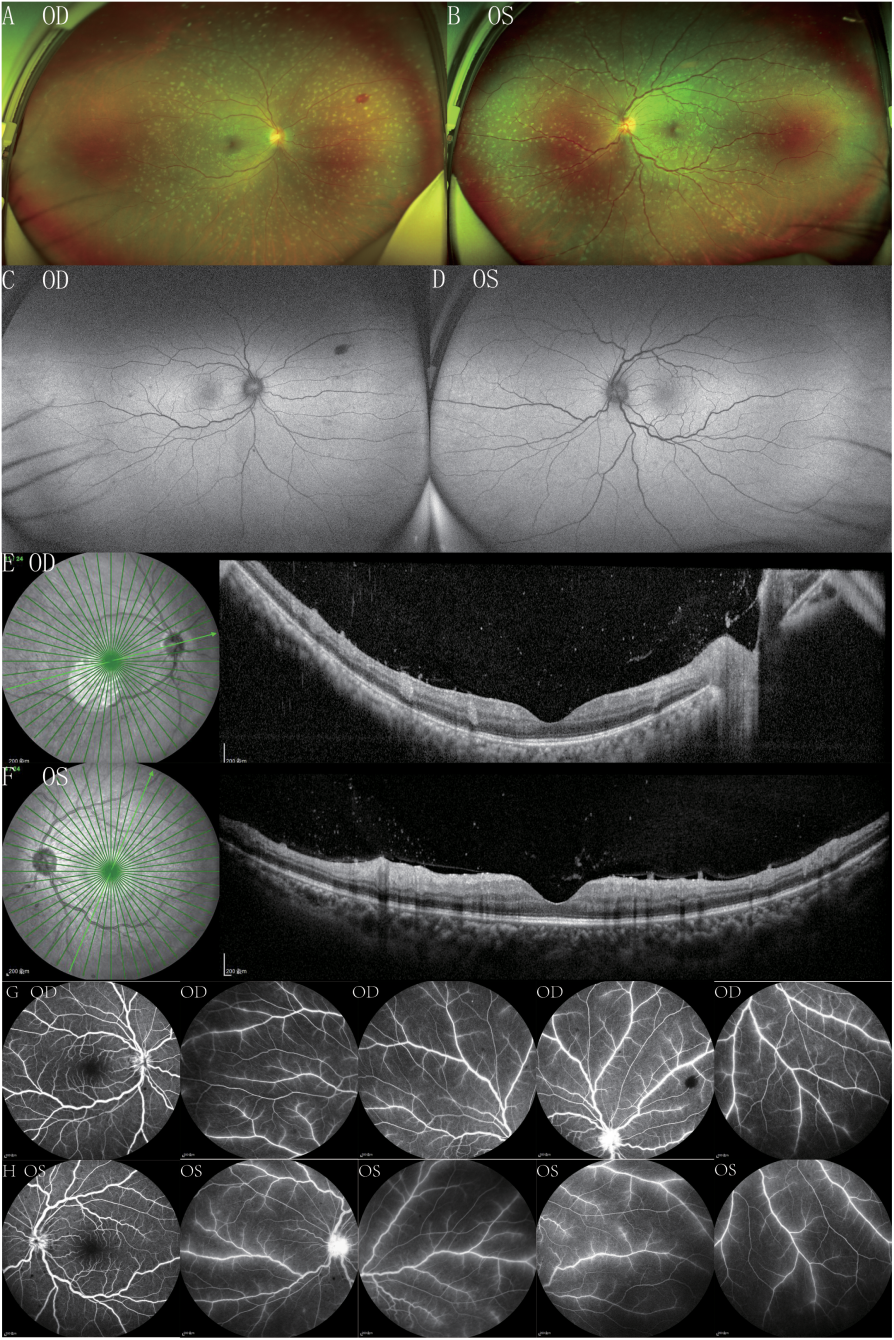
Color fundus photographs of the right eye **(A)** and the left eye **(B)** demonstrate disc edema and diffuse white punctate lesions across the entire retina, mainly distributed along the retinal blood vessels, along with tortuous retinal blood vessels and an unclear reflection of the macular fovea. FAF revealed no significant abnormalities in the right eye **(C)** and the left eye **(D)**. OCT showed that the lesions were distributed in dot, cluster, and mass shapes, located at the junction of the retina and the vitreous, accompanied by inner - layer retinal edema in the right eye **(E)** and the left eye **(F)**. FFA of the right **(G)** and left **(H)** eyes showed vascular fluorescein leakage.

**Figure 2:**
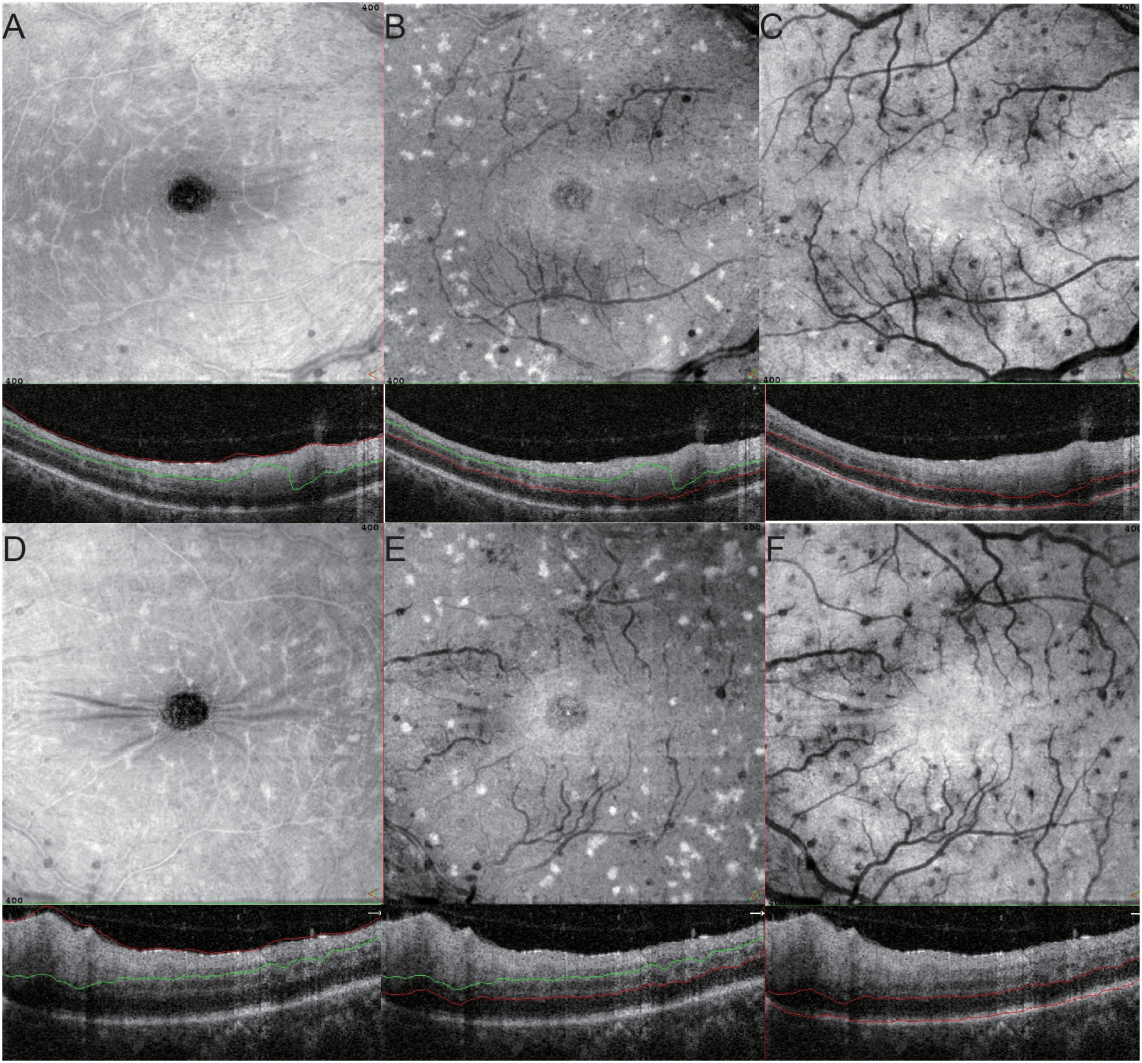
The punctate lesions on En - face OCT in the right eye are distributed in the superficial **(A)**, deeper **(B)**, and outer **(C)** retinal layers of the retina. The punctate lesions on En - face OCT in the left eye are distributed in the superficial **(D)**, deeper **(E)**, and outer **(F)** retinal layers of the retina.

On the fourth day of hospitalization, she consulted the ophthalmology clinic due to a two-day history of blurred vision in both eyes. Best corrected visual acuity (BCVA) was 20/25 in the right eye (OD) and 20/30 in the left eye (OS). Intraocular pressure was normal in both eyes (OU). Slit-lamp examination showed no obvious abnormalities in the anterior segment. Fundus examination disclosed optic disc edema with blurred boundaries, tortuous retinal blood vessels, scattered patchy hemorrhages, and scattered white spot-like lesions along the blood vessels (**Figure 1**). Fundus Autofluorescence (FAF) showed no significant abnormalities (**Figure 1**). Spectral domain optical coherence tomography (SD-OCT) indicated retinal edema, with punctate and clustered high reflexes on the surface of the retina and within the vitreous (**Figure 1**). Fundus fluorescein angiography (FFA) showed fluorescein leakage of retinal blood vessels in both eyes accompanied by late stage high fluorescence of the optic disc, indicating damage to the blood retinal barrier (**Figure 1**). Enface OCT revealed white punctate lesions throughout the superficial, middle, and deep retinal layers in both eyes (**Figure 2**).

A diagnosis of bilateral retinal vasculitis was initially made, prompting cranial magnetic resonance imaging (MRI) and optic nerve MRI scans, which showed no abnormalities. The detection of IgM antibodies against respiratory tract pathogens in serum revealed positive results for Legionella pneumophila IgM antibody. Peripheral blood next-generation sequencing (NGS) revealed no significant abnormalities, but cerebrospinal fluid NGS identified two Legionella sequences. Autoimmune disease markers, including anti-streptolysin O(ASO), rheumatoid factor (RF), four types of immunoglobulins (IgA/IgG/IgM/IgE), human leukocyte antigen B27(HLA-B27) assay, anti-nuclear antibody spectrum, and antineutrophil cytoplasmic antibody assay (ANCA) were normal. After excluding autoimmune diseases such as systemic lupus erythematosus, Behcet’s disease, and ankylosing spondylitis, the systemic diagnosis leaned towards cytokine storm syndrome, potentially induced by Legionella infection. To further elucidate the nature of intraocular lesions, intraocular fluid (aqueous humor) examination was performed, revealing increased inflammatory factors but no other significant abnormalities (Table 1).

Based on fundus manifestations, intraocular fluid testing, imaging results, and systemic examination findings, we attributed the ocular lesions primarily to systemic inflammation-induced intraocular inflammatory responses. Consequently, the patient received bilateral paraocular dexamethasone injections (20 mg each). She also underwent symptomatic supportive treatment with antibiotics (levofloxacin, doxycycline), antiviral medication (acyclovir), and fluid replacement. The patient was discharged after three weeks of improvement and continued follow-up at the ophthalmology clinic.

**Figure 3:**
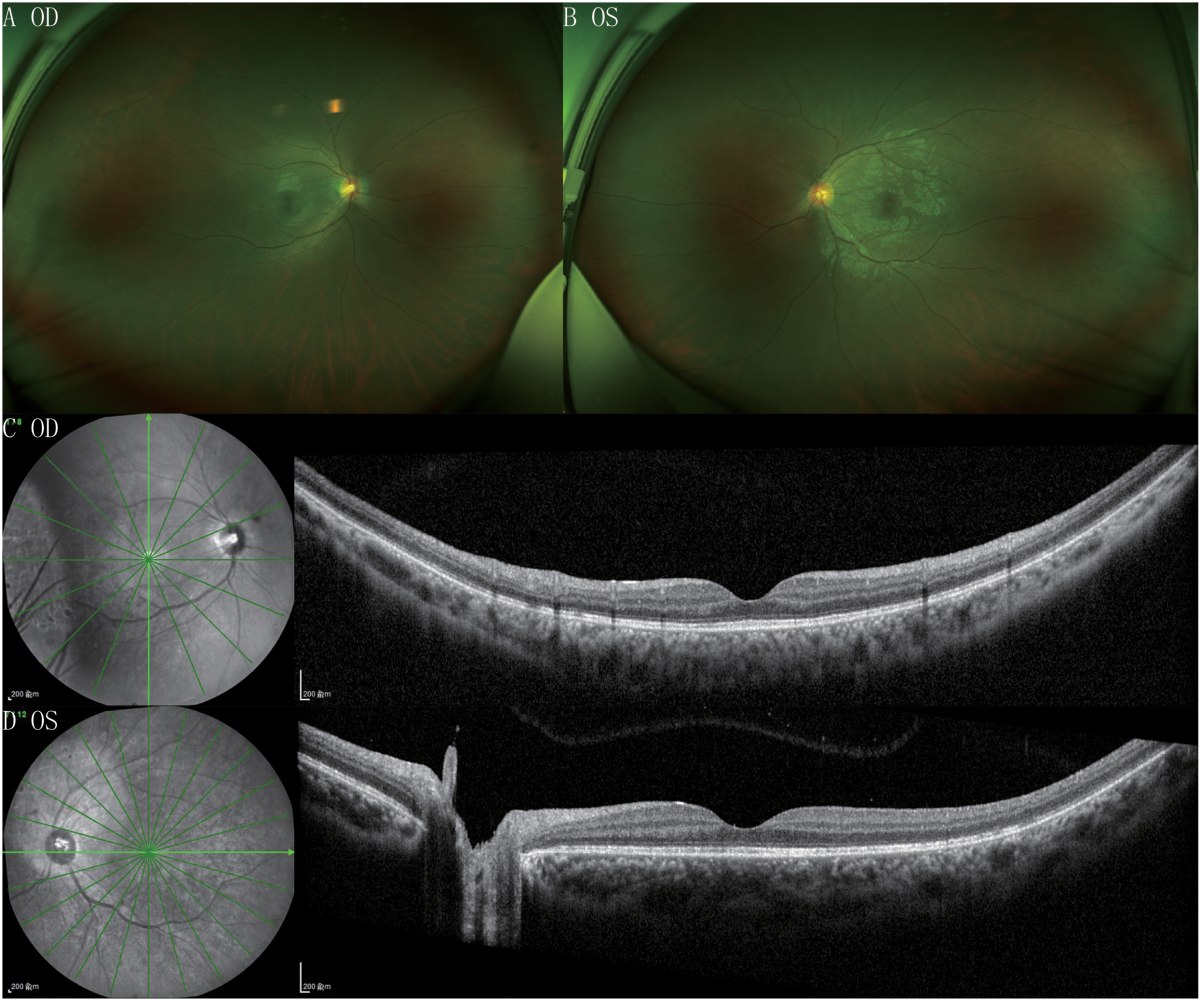
This is an image of the patient during the recovery period. The retinal images are shown as **(A)** (right) and **(B)** (left). **(C)** (right) and **(D)** (left) are OCT examination images.The images all show the disappearance of punctate lesions.

One month post-discharge, her visual acuity was 20/25 in both eyes, with no anterior segment abnormalities. Retinal white spot-like lesions had resolved, OCT showed resolved retinal edema (**Figure 3**), and punctate high reflexes on the retinal surface and vitreous had disappeared (**Figure 3**). FFA and Enface OCT results had normalized. FFA showed no fluorescein leakage, optic disc fluorescence was normal, and Enface OCT showed disappearance of patchy lesions in each layer of the retina in both eyes.

## Discussion

Cytokine storm syndrome (CSS) can be defined as a life-threatening systemic inflammatory state characterized by elevated levels of circulating cytokines and overactivation of immune cells, which can be triggered by infectious or malignant tumors, autoimmune and autoinflammatory conditions. Clinically, CSS is categorized into several types based on its etiology: familial hemophagocytic lymphohistiocytosis, infection-related cytokine storm syndrome, rheumatic cytokine storm syndrome, malignant-tumor-related hemophagocytic lymphohistiocytosis. Infection-related cytokine storm syndrome is commonly characterized by high ferritin levels, elevated CRP, liver and gallbladder dysfunction, coagulation disorders, and thrombocytopenia, and is associated with elevated LDH and D-dimer levels (2, 3). The diagnosis of CSS hinges on the identification of clinical features, laboratory findings, and potential triggering factors. Characteristic symptoms encompass persistent fever, fatigue, headache, cytopenias, and elevated inflammatory markers, including cytokines, CRP, and ferritin (3). In this case, upon admission, the patient exhibited clinical symptoms such as fever, fatigue, headache, and diffuse painful maculopapules on the lower extremities. Before admission, the community hospital conducted tests revealing decreased white blood cell count and platelet count. After admission, laboratory tests showed elevated CRP levels, abnormal transaminase levels, along with elevated levels of IL-6 and PCT in the blood, and a decreased absolute value of NK cells. The patient’s serum tested positive for Legionella pneumophila IgM antibody, and NGS detected two Legionella pneumophila sequences in the cerebrospinal fluid. After ruling out autoimmune diseases, based on the aforementioned evidence, we consider the diagnosis of an infection-related cytokine storm to be appropriate.

In the context of the ongoing COVID-19 pandemic, it has become evident that infections can precipitate fatal cytokine storms in susceptible individuals. Patients succumbing to COVID-19 often exhibit erythropenia, encompassing lymphopenia, anemia, and thrombocytopenia, along with significantly elevated aspartate aminotransferase, D-dimer, and lactate dehydrogenase levels, as well as marked hyperproteinemia. Furthermore, serum levels of IL-6, IL-8, and tumor necrosis factor are notably higher in severe COVID-19 cases compared to healthy blood donors and CAR patients without signs of cytokine release syndrome (CRS) (4–7).

During the COVID-19 pandemic, widespread viral infections have led to the dissemination of cytokine storm syndrome, with ocular involvement being increasingly reported. Proinflammatory mediators produced during cytokine storms can systemically activate the coagulation pathway, resulting in microthrombosis, capillary occlusion, vascular leakage, cell dysfunction, and subsequent tissue ischemia (4, 8). Vagner Loduca Lima et al. reported that among 50 COVID-19 patients in 2020, 7 (14%) experienced eye symptoms, 3 (6%) had temporary low vision, and 4 (8%) reported post-ocular pain (9). Landecho et al. evaluated 27 asymptomatic COVID-19 patients and found that 6 (22%) had obvious cotton exudate (CWS), indicating that CWS is the primary form of ocular vascular damage in COVID-19, potentially related to the direct effects of the virus and its high immunogenicity, leading to endothelial inflammation(similar to vasculitis) or thrombosis (10).

In the case under discussion, the patient demonstrated a significant elevation in serum IL-6 levels, accompanied by markedly increased levels of IL-6, IL-8, and VCAM in the intraocular fluid. These molecules are all pro-inflammatory cytokines implicated in CSS. IL-6, a pleiotropic cytokine, exerts profound effects on immune responses and inflammation through diverse pathways. Its primary roles in the eye include promoting angiogenesis and inducing ocular inflammation. IL-6 signaling pathways are pivotal in various retinal diseases, such as diabetic retinopathy, uveitis, age-related macular degeneration, glaucoma, retinal vein occlusion, central serous chorioretinopathy, and proliferative vitreoretinopathy (11). Within the retina, macrophages and microglia serve as significant sources of IL-6 production. IL-6 transduction mediates inflammation, apoptosis, and barrier disruption in retinal endothelial cells. Inhibiting the IL-6 signaling pathway with sgp130 Fc can alleviate vascular inflammation and endothelial barrier disruption (12). The concentrations of interleukin-6 (IL-6), interleukin-8 (IL-8), monocyte chemoattractant protein-1 (MCP-1), and vascular endothelial growth factor (VEGF) in the vitreous humor of patients with proliferative diabetic retinopathy (PDR) and Eales’ disease were significantly elevated compared to those in patients with macular holes. This suggests that IL-6 play a pivotal role in inflammatory responses and pathological angiogenesis (13, 14). In patients with Behçet’s disease complicated by active retinal vasculitis, aqueous humor levels of IL-6 and IL-8 were markedly higher than those in healthy controls. Following intravitreal methotrexate (MTX) administration, these cytokine levels decreased significantly in parallel with the resolution of intraocular inflammation. These findings suggest that ocular IL-6 and IL-8 levels in Behçet’s disease patients may serve as biomarkers closely associated with retinal vasculitis activity (15). modeling Intravitreal injection of angiotensin II elevates IL-6 expression in the retina and significantly enhances white blood cell adhesion to the vascular wall, an effect completely abrogated in IL-6-deficient mice (16). Vascular cell adhesion molecule (VCAM-1), an inducible transmembrane glycoprotein predominantly expressed in endothelial cells, shows significantly increased expression in ischemic retinas. Overexpression of VCAM-1 protein leads to dysfunction of retinal vascular endothelial cells. Adhesion molecules such as intracellular adhesion molecule-1 (ICAM-1) and VCAM-1 are considered primary drivers of neovascularization in diabetic retinopathy(DR) (17). In experimental non-infectious uveitis, VCAM-1 is expressed on all blood-retinal barrier cells and plays a crucial role in recruiting inflammatory cells during disease progression (18). Moreover, VCAM-1 is most strongly expressed on the inner blood-retinal barrier (BRB), while ICAM-1 is predominantly expressed on the outer BRB in autoimmune uveitis, representing distinct entry pathways for inflammatory cells into the eye (19).

In the early stages of the disease, the patient’s bilateral OCT revealed dot-shaped and clustered high reflexes at the retina-vitreous interface. Enface OCT examination showed that these dot-shaped and clustered lesions were located throughout the entire retina, while FFA showed fluorescein leakage, indicating the destruction of the BRB. The concentrations of inflammatory factors, including IL-6, IL-8, VCAM, BFGF, are elevated in the intraocular fluid. This observation suggests that the punctate and extensive hyperreflective foci at the vitreoretinal interface and within the retina are associated with active intraocular inflammation. Given the resolution limitations of OCT instruments, the term “vitreoretinal interface” refers to the 5 - 10-micron zone located at the actual vitreoretinal interface (20). Transparent cells are a special type of resident macrophages in the vitreous. Microglia are macrophages primarily located in the inner and outer plexiform layers of the normal retina. A smaller proportion is also present in the ganglion cell layer, nerve fiber layer, and near the inner limiting membrane. Large retinal blood vessels pass through the nerve fiber layer of the retina, directly beneath the inner limiting membrane (ILM). Along these blood vessels, there exists a unique anatomical space where perivascular macrophages reside. In research, transparent cells, microglia, and perivascular macrophages are collectively termed macrophage-like cells (MLCs) (20–22). These cells play a pivotal role in retinal immune defense by performing a wide range of functions, from antigen presentation to phagocytosis, thereby ensuring the structural and functional integrity of the retina. When inflammation occurs, the BRB is disrupted, and the MLCs are primarily composed of monocytes and macrophages derived from monocytes. These cells appear as highly reflective bodies on OCT, and are significantly activated during ischemia and disruption of the BRB in the retina. Their number and density increase, and their morphology becomes larger and plumper. When appropriate anti-inflammatory treatment is initiated, MLCs will return to a stable state, with a reduction in quantity, size, and concentration. MLCs play a role in various retinal diseases (20, 22, 23). The increased density and morphological changes of MLCs observed by OCT in retinal vein occlusion (RVO) suggest widespread activation and aggregation of MLCs. OCT imaging of macrophages on the surface of the retina is a potential biomarker for inflammation during RVO (24, 25). Francesco Pichi et al. used surface SS-OCTA to demonstrate an increase in the number and size of vascular retinal inflammatory cells (VRICs) in active uveitis. After inflammation was relieved, the number, size, and density of VRICs significantly decreased (22). The number and density of MLCs in the macular area of DR patients with diabetic macular edema (DME) were significantly higher than those without DME, with larger and fuller morphology (23). The density of MLCs on the ILM is significantly increased in acute non-arteritic anterior ischemic optic neuropathy (NAION) eyes (26). MLCs density increases in Behcet’s uveitis, which can serve as a non-invasive indicator of fluorescein leakage and severity of retinal inflammation in Behcet’s uveitis (27).

After the patient underwent systemic medical treatment, their overall condition improved, and the white punctate lesions on the retina disappeared. Fundus fluorescein angiography (FFA) showed the recovery of the retinal barrier function, and the punctate and massive high reflectivity on optical coherence tomography (OCT) also disappeared accordingly. The patient’s vision also returned to normal.

## Conclusion

We reported a case of retinal-specific manifestations caused by systemic cytokine storm syndrome, which was attributed to elevated circulating cytokine levels and excessive immune cell activation that damaged the ocular vascular barrier, leading to eye involvement, particularly the retina. Its manifestations were captured and documented, highlighting the need for special attention to ocular conditions during cytokine storm syndrome.

## Data Availability

The datasets presented in this study can be found in online repositories. The names of the repository/repositories and accession number(s) can be found in the article/supplementary material.
