# Supplementary material for: Case Report: Cytokine storm syndrome causing retinal inflammatory factor storm
Source: Front Immunol. 2026 May 12;17:1835853. doi: 10.3389/fimmu.2026.1835853 (PMC13201202; doi:10.3389/fimmu.2026.1835853)
Supplement: Supplementary file 2 [file Table2.doc]

March 19, 2026

*Frontiers in Immunology*

Dear Editor:

I wish to submit a case report for publication in *Frontiers in Immunology* , titled “Case Report: Cytokine storm syndrome causing Retinal cytokine storm” .The paper was coauthored by Na Liu, Jingyi Bai, Gaixia Zhai , Shaopeng Wang.

This study reported a young woman with Cytokine storm syndrome who also experienced blurred vision. Upon examination, white punctate lesions resembling " Starry Sky" appeared in the retina, and the lesions completely disappeared after treatment. Whether this sign exists in more patients with systemic inflammation without visual impairment, and what the source of fundus lesions is, have prompted our thinking.

Further, we believe that this paper will be of interest to the readership of your journal because The fundus changes in this case are very rare and closely related to multiple disciplines (such as respiratory, infectious, surgical, etc.), and are worthy of being seen by more disciplines and experts.

This manuscript has not been published or presented elsewhere in part or in entirety and is not under consideration by another journal. We have read and understood your journal’s policies, and we believe that neither the manuscript nor the study violates any of these. There are no conflicts of interest to declare.

Thank you for your consideration. I look forward to hearing from you.

Sincerely,

Shaopeng Wang

Zibo Central Hospital

Department of ophthalmology

China

Tel.:18678186675

Email: [904268100@qq.com](mailto:904268100@qq.com)
